# *Peucedanum japonicum*-Derived Exosome-like Nanovesicles Alleviate Contact Dermatitis

**DOI:** 10.3390/cimb47110909

**Published:** 2025-11-01

**Authors:** Yusuke Yamazumi, Tomoatsu Hayashi, Takuya Kojima, Takeaki Oda, Yasunari Kageyama, Tsutomu Nakamura, Yuki Kamoshida, Tetsu Akiyama

**Affiliations:** 1 Laboratory of Molecular and Genetic Information, Institute for Quantitative Biosciences, The University of Tokyo, Bunkyo-ku, Tokyo 113-0032, Japanhiroton-h@iqb.u-tokyo.ac.jp (T.H.); takeaki@iqb.u-tokyo.ac.jp (T.O.); kamo110@iqb.u-tokyo.ac.jp (Y.K.); 2 Takanawa Clinic, Minato-ku, Tokyo 108-0074, Japan; 3 Tokai University Hospital, Isehara-shi 259-1193, Japan

**Keywords:** exosome-like nanovesicles, inflammation, dermatitis, IL-1β, CXCL2

## Abstract

Contact dermatitis is a common inflammatory skin disorder triggered by exposure to allergens or irritants and characterized by erythema, swelling, and immune cell infiltration. In this study, we investigated the anti-inflammatory effects of exosome-like nanovesicles derived from *Peucedanum japonicum* Thunb. (PjELNs) using a 2,4,6-trinitrochlorobenzene (TNCB)-induced mouse model of contact dermatitis. Intraperitoneal administration of PjELNs markedly reduced ear swelling and histopathological damage and decreased infiltration of inflammatory immune cells, particularly neutrophils. Moreover, PjELNs downregulated the expression of key pro-inflammatory cytokines, including CXCL2 and IL-1β, in the affected tissue. These findings indicate that PjELNs alleviate contact dermatitis-associated inflammation and suggest their potential as a novel plant-derived therapeutic modality for inflammatory skin diseases.

## 1. Introduction

Contact dermatitis is a common inflammatory skin disorder triggered by exposure to allergens or irritants, and is characterized by erythema, swelling, and infiltration of immune cells at the site of exposure [1,2]. Contact dermatitis is generally classified into two subtypes: allergic contact dermatitis, a delayed-type hypersensitivity reaction mediated by allergen-specific T cells, and irritant contact dermatitis, an acute or chronic inflammatory condition caused by direct irritation of the skin by environmental factors such as chemicals or physical, and biological agents. Allergic contact dermatitis progresses through a sensitization phase followed by an elicitation phase upon re-exposure [3,4].

At the molecular level, contact dermatitis involves a complex interplay between keratinocytes, dendritic cells, T cells, and infiltrating leukocytes. Upon exposure to irritants or allergens, keratinocytes release cytokines and chemokines such as IL-1β, TNF-α, IL-6, CXCL1, and CXCL2, which promote leukocyte recruitment and amplify inflammation. In allergic contact dermatitis, Th2 cytokines including IL-4 and IL-13 further enhance immune activation and tissue remodeling. These inflammatory mediators together contribute to skin swelling, redness, and barrier disruption, underscoring their importance as therapeutic targets in contact dermatitis [3,5,6,7].

Due to its well-characterized immune mechanisms, contact dermatitis is widely used as a model to study immune-mediated skin inflammation and to identify therapeutic targets for chronic inflammatory conditions [5,6].

Extracellular vesicles (EVs) are lipid bilayer-enclosed particles secreted by cells that facilitate intercellular communication [8,9]. These vesicles, including exosomes, microvesicles, and apoptotic bodies, carry a variety of biomolecules such as proteins, lipids, and RNAs, and are capable of modulating diverse biological processes in recipient cells. EVs have been implicated in immune regulation, tissue repair, and disease progression, and are increasingly explored as therapeutic agents, particularly in inflammatory and neoplastic diseases [10,11]. Their ability to deliver bioactive cargo selectively makes EVs attractive candidates for drug delivery and disease diagnostics [12,13,14].

We previously reported that exosome-like nanovesicles isolated from *Peucedanum japonicum* Thunb. (PjELNs), a perennial herb in the Apiaceae family, exhibit potent anti-inflammatory effects in a DSS-induced mouse model of colitis [15]. Given these findings, we investigated the efficacy of PjELNs using TNCB-induced mouse model of contact dermatitis, as previously established [16].

## 2. Materials and Methods

### 2.1. Animals

Male Balb/c mice were purchased from Charles River (Yokohama, Japan). Mice were randomly divided into three groups (Non-inflamed (PBS only): n = 3; TNCB + PBS: n = 6; TNCB + PjELNs: n = 6) to ensure comparable body weights and used for the PjELNs treatment experiments at 8 to 11 weeks of age. Mice were maintained in a temperature-controlled room with a 12 h light-dark cycle and allowed free access to food and water under specific pathogen-free conditions at the Institute for Quantitative Biosciences (Tokyo, Japan). All animal experiments were performed according to the Guidelines for the Care and Use of the Institute for Quantitative Biosciences, the University of Tokyo (approval numbers A2022IQB004-03, 30 July 2025). The sample size was determined based on previous studies using a similar model, ensuring sufficient power to detect biologically relevant differences.

### 2.2. Preparation of PjELNs

*Peucedanum japonicum* Thunb. was purchased from a local grocery store (Okinawa, Japan) and stored at 4 °C until use. PjELNs was prepared as previously described [15]. Briefly, the leaves of *Peucedanum japonicum* Thunb. crushed with a juicer were centrifuged at 3000× *g* for 20 min and then at 10,000× *g* for 40 min to remove debris. EVs were pelleted by ultracentrifugation at 150,000× *g* for 90 min, resuspended in PBS, and separated on a sucrose density gradient consisting of 60%, 45%, 30%, and 8% sucrose solutions. Ultracentrifugation was repeated, and fractions at 30–45% (Band2) and 8–30% (Band1) sucrose density boundaries were collected, pelleted, and resuspended. Particle size and concentration were measured via Nanoparticle Tracking Analysis (NTA) with a NanoSight NS300 system (Malvern Instruments, Malvern, Worcestershire, UK) equipped with a 405 nm blue laser. NTA determines the size and concentration of nanoparticles by tracking the Brownian motion of individual particles in suspension using a laser-illuminated microscope and a video analysis system. The analysis was performed according to the manufacturer’s instructions. EVs were stored at 4 °C or −80 °C. The characterization of PjELNs, including NTA and transmission electron microscopy (TEM), was performed as described previously [15], confirming particle size, concentration, and morphology.

### 2.3. Allergic Contact Dermatitis Model

**Figure 1:**
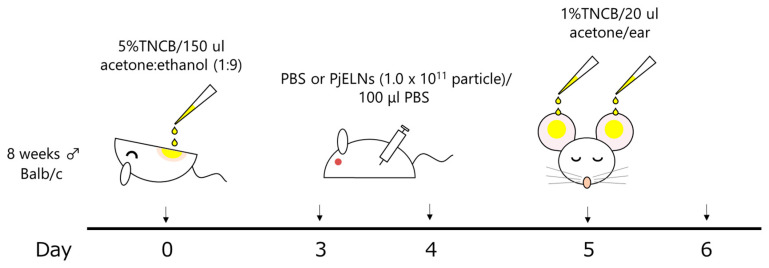
Schematic representation of the experimental protocol for the TNCB-induced contact dermatitis model. Male (♂) mice were sensitized with TNCB on the abdomen on day 0, treated with PjELNs or PBS on days 3 and 4, and challenged with TNCB on the ears on day 5. Ear thickness and tissue analyses were performed 24 h after challenge.

Eight-week-old male Balb/c mice were sensitized on day 0 by applying 150 μL of 5% (*w*/*v*) 2,4,6-trinitrochlorobenzene (TNCB; Tokyo Chemical Industry, Tokyo, Japan) in a 1:9 acetone solution to their shaved abdominal skin [16]. On days 3 and 4, PjELNs (1.0 × 10^11^ particles/100 μL PBS per mouse) or PBS (100 μL per mouse) were administered intraperitoneally. On day 5, 10 μL of 1% TNCB in acetone was applied to both sides of each ear to induce contact dermatitis; acetone alone was used for the non-inflammation group. Ear thickness was measured with a micrometer after 24 h after TNCB application, and the average of two measurements was recorded as each mouse’s ear thickness. Subsequently, cells from the right ear were collected for FACS analysis, and RNA from the left ear for qPCR. The remaining left ear tissue was fixed in 10% formalin/PBS, embedded in paraffin, and sectioned for H&E staining. The experimental design of the TNCB-induced contact dermatitis model is illustrated in Figure 1.

### 2.4. Histological Staining

H&E staining was performed as previously described [15]. Briefly, after perfusion with PBS, the ears were fixed overnight in 4% PFA. After fixation, the samples were dehydrated in a series of ethanol solutions (20%, 40%, 60%, 80%, 100%) and subsequently substituted with xylene before being embedded in paraffin. Sections of 8 μm thickness were cut from the paraffin blocks using a microtome. The sections were deparaffinized in xylene and then rehydrated through a series of ethanol solutions (100%, 80%, 60%, 40%, 20%) before being subjected to morphological observations using Hematoxylin and Eosin staining.

### 2.5. FACS Analysis

FACS analysis was performed immediately after ear tissue collection. Cells from the epidermis and dermis of the ear skin were dissociated into single-cell suspension using the Multi Tissue Dissociation Kit 1 (Miltenyi, Bergisch Gladbach, Nordrhein-Westfalen, Germany) and the gentleMACS Octo Dissociator with Heaters (Miltenyi). After passing through a 100 μL cell strainer and lysing to remove red blood cells, cells were suspended in MACS Rinsing Buffer (Miltenyi). They were counted, blocked with anti-CD16/32 antibodies, and stained with various markers: anti-CD11b-FITC, anti-F4/80-PE, anti-Ly6G-PE-Cy7, anti-CD3e-APC, and anti-CD45/Cy7 (BioLegend, San Diego, CA, USA). Cell viability was assessed using BD Cell Viability Solution (BD, Franklin Lakes, NJ, USA), and inflammatory cytokine expression was analyzed using the FACS Melody (BD). Data were analyzed using FlowJo (v10) software (BD). Dead cells and debris were excluded by forward/side scatter gating and viability dye. Lymphocytes were gated based on forward and side scatter profiles, and T cells were identified as CD3^+^CD45^+^ populations. Macrophages (CD11b^+^F4/80^+^) and neutrophils (CD11b^+^Ly6G^+^) were also quantified.

### 2.6. RT-qPCR

A portion of the left ear tissue was suspended in Trisure (Bioline, Gregory Hills, NSW, Australia), homogenized and stored for several days at −80 °C. RNA was extracted according to the manufacturer’s protocol, followed by cDNA synthesis using Primescript (TAKARA, Shiga, Japan). qPCR was then performed on the LightCycler 480 using SYBR Green qPCR Master Mix (Roche, Basel, Switzerland) and the following primers: IL-4 forward:5′-CATCGGCATTTTGAACGAG-3′; IL-4 reverse: 5′-CGAGCTCACTCTCTGTGGTG-3′; IL-13 forward: 5′-CCTCTGACCCCTTAAGGAGCTTAT-3′; IL-13 reverse: 5′-CGTTGCACAGGGGAGTCT-3′; CXCL1 forward: 5′-AGACTCCAGCCACACTCCAA-3′; CXCL1 reverse: 5′-TGACAGCCAGCTCATTTG-3′; CXCL2 forward: 5′-AAAATCATCCAAAAGATACTGAACAA-3′; CXCL2 reverse: 5′-CTTTGGTTCTTCCTTGTTGGAGG-3′; IL-33 forward: 5′-GACACATTGAGCATCCAAGG-3′; IL-33 reverse: 5′-AACAGATTGGTCAATTGTAAGTACTCAG-3′; IL-6 forward: 5′-GCTACCAAAGTTGGATATAATCAGGA-3′; IL-6 reverse: 5′-CCAGGTAGCTATGGTTACTCCAGA-3′; IL-1β forward: 5′-AGTTGACGGACCCCAAAAG-3′; IL-1β reverse: 5′-AGCTGGATGCTCTCATCAGG-3′; TNF-α forward: 5′-TCTTCTCATTCCCTGCTTGTGG-3′; TNF-α reverse: 5′-GAGGCCATTTGGGAAGCTTCT-3′.

### 2.7. Statistical Analysis

Quantitative data are expressed as mean ± standard error of the mean (SEM). Comparisons between two groups were conducted using unpaired Student’s *t*-test. In all analyses, a *p*-value < 0.05 was considered statistically significant.

## 3. Results

### 3.1. PjELNs Suppress TNCB-Induced Ear Swelling in Mice

**Figure 2:**
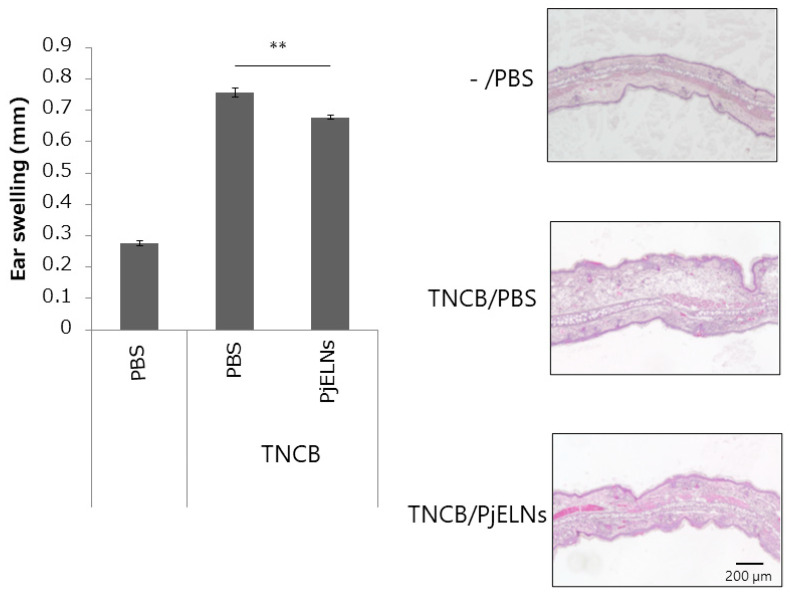
PjELNs suppress TNCB-induced ear swelling. The study included three groups of mice: untreated controls (PBS), mice with TNCB administration only (TNCB/PBS), and mice with TNCB administration followed by PjELNs treatment (TNCB/PjELNs). Ear thickness was measured 24 h after TNCB challenge in mice treated with PjELNs or PBS. PBS: n = 3; TNCB/PBS: n = 6; TNCB/PjELNs: n = 6. Data are shown as mean ± S.E. ** *p* < 0.01, unpaired Student’s *t*-test. Scale bar: 200 μm.

**Figure 3:**
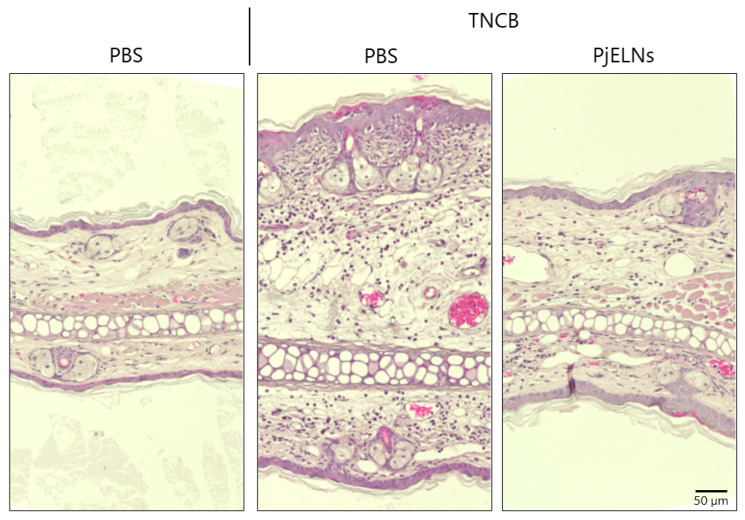
Histological analysis of ear tissue by H&E staining. Ears were collected 24 h after TNCB challenge in mice treated with or without PjELNs. Representative images of ear sections from each treatment group. Non-inflamed (PBS only): n = 1; TNCB + PBS: n = 4; TNCB + PjELNs: n = 4. Scale bar: 50 μm.

The effects of PjELNs were evaluated in the TNCB-induced contact dermatitis model as outlined in Figure 1. TNCB administration significantly increased ear thickness, whereas PjELNs treatment markedly suppressed this effect compared to PBS-treated controls (Figure 2). Histological analysis using H&E staining revealed that TNCB caused epidermal and dermal thickening, structural disruption, and dense inflammatory cell infiltration, which were alleviated by PjELNs treatment (Figure 3).

### 3.2. PjELNs Inhibit TNCB-Induced Inflammatory Cell Infiltration

**Figure 4:**
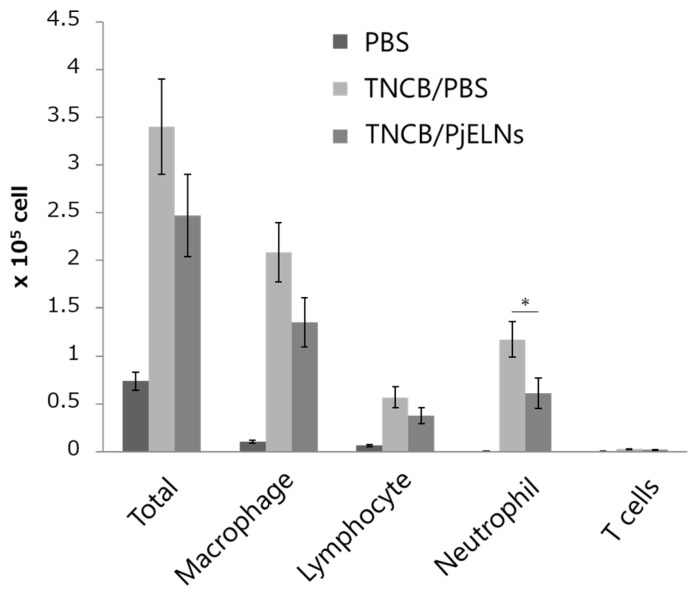
PjELNs reduce TNCB-induced inflammatory cell infiltration. FACS analysis was performed on ear skin cells from each group 24 h after TNCB challenge in mice treated with PjELNs or PBS. PBS: n = 3; TNCB/PBS: n = 6; TNCB/PjELNs: n = 6. Data are presented as mean ± S.E. * *p* < 0.05, unpaired Student’s *t*-test.

To evaluate immune cell infiltration, single-cell suspensions from ear tissues were analyzed by flow cytometry. PjELNs treatment significantly reduced the number of neutrophils, while other inflammatory cell populations showed a decreasing trend without statistical significance (Figure 4), suggesting that PjELNs suppress TNCB-induced leukocyte infiltration, particularly neutrophils.

### 3.3. PjELNs Suppress TNCB-Induced Expression of Pro-Inflammatory Cytokines

**Figure 5:**
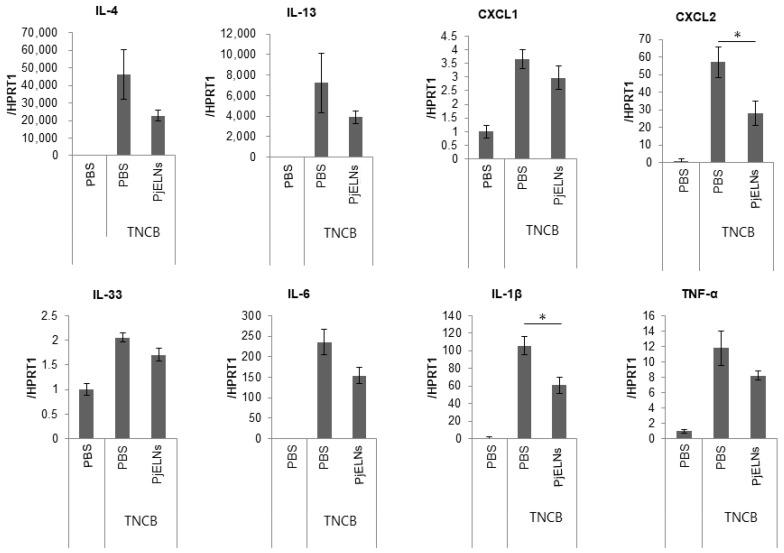
PjELNs reduce expression of inflammatory cytokines in TNCB-administrated skin. qPCR analysis of cytokine mRNA levels in ear skin tissue from each group 24 h after TNCB challenge in mice treated with or without PjELNs. PBS: n = 3; TNCB/PBS: n = 6; TNCB/PjELNs: n = 6. Data are presented as mean ± S.E. * *p* < 0.05, unpaired Student’s *t*-test.

Quantitative PCR analysis of ear tissue revealed that TNCB application upregulated the expression of multiple pro-inflammatory cytokines, including IL-4, IL-13, CXCL1, CXCL2, IL-33, IL-6, IL-1β, and TNF-α (Figure 5). Treatment with PjELNs significantly attenuated the expression of these cytokines, particularly CXCL2 and IL-1β. These findings suggest that PjELNs attenuate cytokine responses associated with neutrophil recruitment and tissue inflammation, mainly through the suppression of CXCL2 and IL-1β.

## 4. Discussion

In this study, we demonstrated that PjELNs exert robust anti-inflammatory effects in a TNCB-induced contact dermatitis model. PjELNs reduced ear swelling, histopathological damage, and inflammatory cell infiltration, especially of neutrophils. Moreover, PjELNs significantly reduced the expression of key inflammatory cytokines and chemokines, particularly CXCL2 and IL-1β, and showed a downward trend in several others, including IL-4, IL-13, and TNF-α. These results are consistent with our previous findings that PjELNs attenuate mucosal inflammation in DSS-induced colitis models by modulating immune cell activation and cytokine production [15]. IL-1β and CXCL2 have been demonstrated to play pivotal roles in the pathogenesis of dermatitis [17,18]. In fact, deficiency or inhibition of IL-1β or CXCR2 has been reported to suppress the onset of dermatitis [19,20,21]. Moreover, the pronounced suppression of CXCL2 and IL-1β by PjELNs in this study likely accounts for the reduction in neutrophil infiltration, which is a key driver of tissue damage and symptom severity in allergic contact dermatitis [21,22,23,24,25].

In our previous study, we demonstrated that PjELNs directly regulate inflammatory cytokine expression through the transfer of small RNAs with miRNA-like activity [15]. These small RNAs were shown to interact with mammalian transcripts involved in inflammatory signaling pathways, suggesting a cross-kingdom regulatory mechanism underlying the anti-inflammatory effects of PjELNs. Although RNA-mediated effects may represent one of the key mechanisms underlying the anti-inflammatory action of PjELNs, other bioactive constituents derived from *Peucedanum japonicum* Thunb., such as coumarins, flavonoids, and chlorogenic acid derivatives, could also contribute in part to the observed activity [26,27]. Together, these findings suggest that both RNA-based and phytochemical components synergistically underlie the biological activity of PjELNs. This notion is consistent with recent reports describing the immunomodulatory roles of plant-derived extracellular vesicles carrying functional small RNAs and secondary metabolites that modulate host immune responses [28,29,30,31].

Given that plant-derived extracellular vesicles are biocompatible and can be readily administered [30,31], PjELNs may serve as a promising therapeutic strategy for inflammatory skin conditions. Further studies are warranted to elucidate their mechanisms of immune regulation, optimize dosage and delivery, and evaluate efficacy in chronic dermatitis models and human patients.

## Data Availability

The original contributions presented in this study are included in the article material. Further inquiries can be directed to the corresponding author.
